# Perinatal development of central vestibular neurons in mice

**DOI:** 10.3389/fnins.2022.935166

**Published:** 2022-09-01

**Authors:** Christophe J. Dubois, Laura Cardoit, John Simmers, François M. Lambert, Muriel Thoby-Brisson

**Affiliations:** Univ. Bordeaux, CNRS, EPHE, INCIA, UMR 5287, Bordeaux, France

**Keywords:** vestibular neuron, lateral vestibulospinal tract (LVST), vestibular commissural pathway, perinatal development, mouse model

## Abstract

Central circuitry of the vestibular nuclei integrates sensory inputs in the adaptive control of motor behaviors such as posture, locomotion, and gaze stabilization. Thus far, such circuits have been mostly examined at mature stages, whereas their emergence and early development have remained poorly described. Here, we focused on the perinatal period of murine development, from embryonic day E14.5 to post-natal day P5, to investigate the ontogeny of two functionally distinct vestibular neuronal groups, neurons projecting to the spinal cord via the lateral vestibulospinal tract (LVST) and commissural neurons of the medial vestibular nucleus that cross the midline to the contralateral nucleus. Using transgenic mice and retrograde labeling, we found that network-constitutive GABAergic and glycinergic neurons are already established in the two vestibular groups at embryonic stages. Although incapable of repetitive firing at E14.5, neurons of both groups can generate spike trains from E15.5 onward and diverge into previously established A or B subtypes according to the absence (A) or presence (B) of a two-stage spike after hyperpolarization. Investigation of several voltage-dependent membrane properties indicated that solely LVST neurons undergo significant maturational changes in their electrophysiological characteristics during perinatal development. The proportions of A *vs* B subtypes also evolve in both groups, with type A neurons remaining predominant at all stages, and type B commissural neurons appearing only post-natally. Together, our results indicate that vestibular neurons acquire their distinct morpho-functional identities after E14.5 and that the early maturation of membrane properties does not emerge uniformly in the different functional subpopulations of vestibulo-motor pathways.

## Introduction

The neuronal circuits of the vestibular system are involved in integrating and transforming sensory vestibular inputs into *ad hoc* fast corrective motor actions. This complex central network is anatomically and functionally subdivided into distinct neuronal populations that by processing a wide variety of signals from primary vestibular afferents, somatosensory organs and the cerebellum, generate sensorimotor reflexes that play an essential role in maintaining equilibrium, posture, head position, and visual acuity during movement. Of the four major constituent nuclei, neurons located in the lateral vestibular nucleus (LVN) are amongst the first vestibular neuronal populations to be established during embryogenesis (Altman and Bayer, 1980). In particular, neurons projecting to the spinal cord through the lateral vestibulospinal tract (LVST) receive inputs from vestibular sensory neurons by E11.5 (Maklad et al., 2010) and reach cervical levels by E12 (Auclair et al., 1999). Although mainly located in the LVN, the somata of LVST neurons are also found in the descending VN (DVN) and in the magnocellular part of the medial vestibular nucleus (MVN) (Straka et al., 2005). The axons of LVST neurons target motoneurons and interneurons located at cervical, thoracic, and lumbar levels in the spinal cord and control vestibulospinal reflexes governing the axial and limb musculature [(Wilson and Peterson, 1978; Wilson et al., 1995; Shinoda et al., 2006; Kasumacic et al., 2010, 2015; Lambert et al., 2016); also see below]. Located more medially, neurons of the MVN are established later in the development, by E14 (Altman and Bayer, 1980), and represent the largest and best studied vestibular neuron population. The MVN is also the largest source of mid-line crossing (commissural) connectivity within the vestibular system (Bagnall et al., 2007). MVN neurons form several functional subgroups that project *via* internuclear neurons to specific sets of motoneurons in contralateral extraocular motor nuclei through inhibitory commissural projections (Mano et al., 1968; Straka et al., 2005; Bagnall et al., 2007; Malinvaud et al., 2010).

The integrative cellular and network properties of vestibular premotor circuitry are relatively well known in adult mammals. In rodents, two types of central vestibular neurons have been characterized according to their electrophysiological profiles [for review see Straka et al. (2005), and Serafin et al. (1991), Dutia and Johnston (1998), Saito and Ozawa (2007), Beraneck and Idoux (2012), Beraneck and Lambert (2020)]. Type A neurons exhibit a monophasic after-spike hyperpolarization (AHP) with an I_*A*_-like K+ current delaying the repolarization during the inter-spike interval. In contrast type B neurons exhibit a biphasic AHP without involving an I_*A*_-like K+ conductance. These two cell types also differ in their action potential (AP) profiles, discharge dynamics and the membrane conductances expressed (Gallagher et al., 1985; Serafin et al., 1991; Johnston et al., 1994; Beraneck et al., 2003; Uno et al., 2003; Bagnall et al., 2007). In addition to specific potassium and calcium conductances, vestibular neurons also possess the persistent sodium current [INaP; (Serafin et al., 1991; Gittis and du Lac, 2008)]. This current is implicated in spontaneous neuronal activity and is likely to be involved in post-inhibitory rebound (PiR), the development of a transient increase in cell excitability at the offset of inhibitory synaptic events (Sekirnjak and du Lac, 2002). The hyperpolarization-activated conductance (Ih) is also likely to play an important role in determining vestibular neuron firing (Zhang et al., 2013; Li et al., 2016) by contributing directly to PiR (Sekirnjak and du Lac, 2002).

Intriguingly, the respective proportions, membrane properties, and discharge patterns of type A and B vestibular neurons evolve during late developmental processes at post-natal stages or after a unilateral labyrinthectomy (Johnston et al., 1994; Dutia and Johnston, 1998; Him and Dutia, 2001; Beraneck et al., 2003; Camp et al., 2006), with at least some of these changes being regulated by sensory vestibular information (Eugène et al., 2007). However, because most current knowledge on vestibular neuron membrane properties has been gained by investigating the mature vestibular system network, the initial developmental acquisition of electrophysiological properties required for the proper integration of vestibular inputs from birth remains poorly examined. Moreover, whether the early maturational processes proceed uniformly before and after birth and across different functional vestibular groups remains unknown.

Here, to address these issues, we employed retrograde tracing on mice to identify distinct neuronal groups according to their axonal projections. Specifically, we focused on LVST neurons that represent one of the main vestibular subpopulations that project outside the vestibular nuclei complex and are responsible for directly governing motor output involved in postural control (Glover, 1995; Shinoda et al., 2006; Kasumacic et al., 2010, 2015). We also identified neurons of the commissural subpopulation that are responsible for the push–pull organization of the vestibular system, mandatory for the bilateral coordination of vestibular neuronal processing. We then aimed to characterize the emergence and early maturation during the perinatal period (from E14.5 to P5) of neurotransmitter phenotypes (using transgenic mice with fluorescence labeling of GABAergic or glycinergic neurons) and the electrophysiological properties of type A and type B neurons belonging to each of the two vestibular groups. Our findings demonstrate that vestibular neuron properties do not emerge and develop uniformly, either across different functional groups or between the A and B subtypes of each group.

## Materials and methods

### Animals

All experiments were performed on embryos (from stage E14.5 onward) and early post-natal pups (up to P5) from mouse lines bred in our animal facility under standard conditions with a 12/12 h dark/light cycle and food and water provided *ad libitum*. Wild-type mice (Swiss) were initially purchased from Janvier Labs (France) and subsequently bred locally. GABAergic neurons were identified using a GAD67-eGFP mouse line also from a Swiss background (Boeri et al., 2018) whereas glycinergic neurons were identified using a GlyT2-GFP mouse line that was bred on a C57Bl6J background (Zeilhofer et al., 2005). Experimental procedures were in accordance with the University of Bordeaux animal care committee regulations and the European Union directive 2010/63/EU.

### *Ex vivo* brainstem-spinal cord preparations

Embryos were extracted from dams euthanized by cervical dislocation. After decapitation, pontomedullary *in vitro* preparations were isolated from embryos or pups and placed in an oxygen-saturated (95% O2, 5% CO2) artificial cerebrospinal fluid (aCSF) chilled at 4°C and containing (in mM): 128 NaCl, 3 KCl, 25 NaHCO3, 1.2 NaH2PO4, 1 MgSO4, 2.5 CaCl2, 23.3 glucose, 5 HEPES, pH 7.4, as described previously (Lambert et al., 2016).

### Retrograde labeling of vestibular neurons with conjugated dextran amines

**FIGURE 1:**
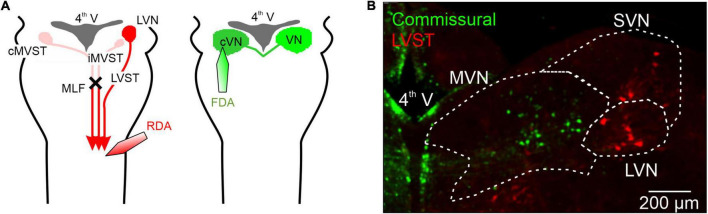
Retrograde labeling of mouse perinatal vestibular neurons with conjugated dextran amines. **(A)** Left, schematic of the protocol for the labeling of the somata of neurons with axons projecting in the lateral vestibulospinal tract (LVST) by using either rhodamine-conjugated dextran amine (RDA) or fluorescein-conjugated dextran amine (FDA). Dye crystals were applied to a unilateral cut made in the ventral and ventrolateral funiculi at the level of spinal C1 in order to label vestibulo-spinal projecting neurons. A bilateral section of the medial longitudinal fasciculus (MLF) was also made to isolate ipsilateral LVST axons from other vestibulo-spinal axons, by preventing dextran amine migration through the axons of ipsilateral and contralateral medial vestibulospinal tracts (iMVST and cMVST). Right, using a similar protocol, brainstem commissural neurons on one side were labeled with RDA or FDA by applying dye crystals to an incision in the contralateral vestibular nuclei. **(B)** Representative double-labeling of commissural (green) and LVST (red) neurons in a 300 μM thick slice from the brainstem of a newborn mouse (at P2). 4th V, fourth ventricle; MVN, medial vestibular nucleus; LVN, lateral vestibular nucleus; SVN, superior vestibular nucleus, MLF, medial longitudinal fasciculus.

Retrograde labeling of LVST and commissural vestibular neurons was obtained using either rhodamine-conjugated dextran amine (RDA, 3 kMW, ThermoFicher, Illkirch, France) or fluorescein-conjugated dextran amine (FDA, 3 kMW, ThermoFicher, Illkirch, France) as previously described (Glover, 1995; Kasumacic et al., 2010). Briefly, crystals of RDA or FDA (R/FDA) were applied to a unilateral cut made in the ventral and ventrolateral funiculi at C1 or the contralateral vestibular nucleus in order to backfill the axons of spino-vestibular or commissural neurons, respectively (Figure 1). In order to differentiate ipsilateral LVST neurons from other spino-vestibular neurons, an additional bilateral section of the medial longitudinal fasciculus (MLF) was made to prevent dextran amine migration in the axons of ipsilateral and contralateral medial vestibulospinal tracts [iMVST and cMVST (Kasumacic et al., 2010)]. Preparations were incubated at room temperature in the dark under aCSF for at least 4 and 3 h for LVST and commissural neuron tracing, respectively.

### Slice preparations

After incubation with dextran amines, preparations were mounted in low-melting agar (4%, ThermoFicher, Illkirch, France) and 350 μm transverse brainstem slices containing the vestibular nuclei were obtained using a vibratome (Leica VS 1000) in ice-cold oxygenated aCSF. Effective labeling of vestibular neurons was assessed using a fluorescence microscope (Nikon, Champigny sur Marne, France). Selected slices were then used for electrophysiology or fixed in 4% PFA overnight for subsequent fluorescence microscopy.

### Electrophysiological recordings and analysis

Patch-clamp recordings were performed exclusively on vestibular neurons that had been identified by retrograde labeling. Experiments were conducted at 35°C using borosilicate patch pipettes (5–8 MΩ) filled with a K-gluconate-based on internal solution (in mM: 140 K-gluconate, 2 MgCl2, 10 HEPES, 3 Na2-ATP, 0.5 Na-GTP and 10 EGTA, pH 7.3). Biocytin (0.02%) was also added to the patch pipette solution to fill recorded neurons and allow their subsequent visualization (see below). Analog signals were filtered at 10 kHz and digitized at 20 kHz (AxoClamp 2B or Multiclamp 700A, Molecular Devices, San Jose, CA, United States). Capacitance, series, and input resistance were monitored using 10 ms, 5 V pulses delivered at patch break-in and after each series of voltage and current clamp experiments. Large variations in any of these parameters led to the termination/rejection of recordings. At the end of each recording session, a mosaic image of the whole slice was taken (10× objective) to determine the recording site location.

#### Current clamp experiments

Neurons that fired APs with amplitude of more than 50 mV, peaked above 0 mV and had an input resistance of at least 100 MΩ were analyzed. Cells with input resistance higher than 100 MΩ and a resting membrane potential more negative than −40 mV but were unable to fire APs were defined as immature neurons. AP frequencies were averaged without current injection whenever possible or at the lowest injected depolarizing current required to elicit firing. AP threshold was defined as the membrane voltage at which the first derivative of the voltage reached 10 V/s prior to an AP. An inflection of the first derivative of the voltage trace during the post-spike repolarization was used to classify recorded neurons as either type A (inflection absent) or type B (inflection present). Firing frequency at rest, AP amplitude and width (at threshold) and AHP amplitude were also quantified.

After a 2-min recording of spontaneous activity, a series of 2 s incrementing pulses with 50 pA steps from −200 to +200 pA were injected, and the average firing frequency during each pulse was measured. The mean instantaneous frequency of the first 5 (initial) and last 5 (final) APs during the 100 pA depolarizing pulse of each series was also used to define the firing rate adaptation ratio (final/initial). The maximal AP frequency immediately after the 200 pA hyperpolarizing pulse was compared to the average frequency before the pulse to evaluate the presence of a PiR property.

#### Voltage clamp experiments

For all series of voltage clamp experiments, recorded vestibular neurons were initially clamped at −50 mV. I/V curves were plotted by measuring the amplitude of the membrane current evoked by 400 ms voltage steps from −100 to +30 mV. Current amplitude was measured at steady state, during the final 50 ms of each step.

The presence of the hyperpolarization-activated current (Ih) was tested by evoking the current, if present, by a series of 2-s voltage steps with increments of 10 mV from −110 to −50 mV. The presence of the persistent sodium current (INaP) was assessed with a voltage ramp protocol from −80 to +40 mV over 9 s. The INaP current was visible as an inward current inflection developing around −40 mV. In some experiments after a first ramp test in control conditions, the fast sodium channel blocker TTX (0.5 μM; Merck, Martillac, France) was added to the bathing aCSF. The TTX-sensitive difference current (corresponding to INaP) was then plotted by digitally subtracting the traces recorded before and after exposure to TTX.

### Fluorescence microscopy

#### Biocytin 3D reconstructions

As mentioned above, the presence of 0.02% of biocytin in the patch pipette solution enabled the precise localization of recorded, dye-filled neurons in each slice as well as their subsequent 3D reconstruction. After completion of recording, slices were removed from the recording chamber and fixed overnight in a PBS solution containing 4% PFA. Following several washes in PBS, sections were incubated at 4°C with streptavidin conjugate (red or green dye concentration: 1:1000) with 0.3% Triton X-100 and 1% BSA in 0.1 M PBS and kept overnight in the dark. After another series of washes, cross-sections were mounted in a homemade mounting medium containing 74.9% glycerol, 25% Coon’s solution (0.1 M NaCl and 0.01 M diethyl-barbiturate sodium in PBS), and 0.1% paraphenylenediamine.

Slices were imaged using a confocal microscope (Olympus FV1000) with 488 or 543 nm lasers, according to the streptavidin conjugate used. Multi-image confocal 4 μm z-stacks were generated using a 20×/0.75 oil objective. After acquisition, the images were processed using Open Source Fiji software. 3D morphology reconstruction, Scholl analysis, and measurements were performed with the Simple Neurite Tracer plugin embedded in Fiji (Longair et al., 2011; Ferreira et al., 2014).

#### Inhibitory neurotransmitter determination

GABAergic and glycinergic neurons within the LVST and vestibular commissural neuronal groups were identified using GAD67-eGFP and GlyT2-GFP mouse lines, respectively. After dextran amine injections as detailed above, *ex vivo* preparations were incubated in oxygenated aCSF at room temperature for 4 h and then fixed in PFA overnight (4% in PBS at 4°C). After a day-long incubation in a 20% sucrose PBS solution at 4°C, the preparations were embedded in a tissue-tek matrix (VWR-Chemicals, Fontenay-sous-Bois, France) and frozen at -45°C using isopentane. 30 μm slices were then cut using a cryostat (CM 3050, Leica, Nanterre, France) and mounted in the homemade medium described above.

Slice imaging was performed using an epifluorescence microscope (Olympus LX-81) with a 10× objective and an ORCA-R2 camera (Hamamatsu, Massy, France). Mosaics of the whole slices were imaged using an automated scanning stage (SCAN IM 112 × 74, Marzhauser, Wetzlar, Germany) controlled by micromanager. Stitching of the final images was made using the Collection Stitching plugin in Fiji (Preibisch et al., 2009). Rotation, alignment, and determination of the distance from *bregma* were performed in CorelDraw according to the *Atlas of the Developing Mouse Brain* (Paxinos et al., 2020).

### Statistics

Fisher’s exact (proportions) and Mann–Whitney tests were used for statistical analyses. All values are reported as mean ± SEM, and differences in mean values were considered to be significant at *p* < 0.05. Except for the “immature” “A” or “B” phenotyping of recorded neurons (Figure 4), biophysical data from all embryonic stages were pooled into a single “pre-natal group” as no significant differences was found between individual stages. Similarly, data from post-natal stages of the two vestibular groups were pooled into single “post-natal groups.”

## Results

Previous studies have described the intrinsic membrane properties of central vestibular neurons, but not in relation to their specific task and only at developmental stages later than P5. In the present study, therefore, we chose to investigate the early development of two identified vestibular neuronal populations–the LVST and the vestibular commissural system–that are known to exhibit distinct functional features. To this end, we focused on two developmental periods: firstly, that leading up to birth (from embryonic stage E14.5), and secondly, the early post-natal period from birth to P5. The soma locations and distributions of the two groups of vestibular neurons were initially determined by selective retrograde labeling with dextran amines (see Section “Materials and methods”). LVST and commissural neurons were labeled by retrograde tracing by dextran amine dye (see Section “Materials and methods”) from their axonal projections in the spinal cord (Figure 1A left) or the contralateral vestibular nucleus (Figure 1A right), respectively. As illustrated by the representative double-stained preparation in Figure 1, spinal-projecting LVST neurons were found mainly in the LVN and the SVN [red fluorescent neurons in Figure 1B; (Glover, 1995; Kasumacic et al., 2010)], whereas somata of commissural neurons were localized mainly in the MVN [green fluorescent neurons in Figure 1B; (Bagnall et al., 2007)], more medially compared to LVST neurons. These tracing procedures, which were systemically conducted in all experiments, thus enabled us to locate the somata of the two different groups of vestibular neurons as a prerequisite for subsequent neurochemical, anatomical, and electrophysiological characterization.

### Perinatal expression of inhibitory neurotransmitter phenotypes

A major differentiating feature of the two vestibular neuronal populations is the extent to which they employ synaptic inhibition. Whereas the vast majority of neurons in the LVST of late post-natal rodents exert excitatory, glutamate-mediated influences on their spinal targets (Du Beau et al., 2012; Basaldella et al., 2015), a large proportion of commissural neurons that send projections to the contralateral MVN are inhibitory, using GABA or glycine as their neurotransmitter (Bagnall et al., 2007; Malinvaud et al., 2010). To assess and compare the extent to which LVST and vestibular commissural neurons express inhibitory transmitters during the perinatal period, we performed selective retrograde labeling (as in Figure 1) on brainstem preparations obtained from two transgenic mice lines, the GAD67-eGFP and GlyT2-GFP models (see Section “Materials and methods”), in order to identify GABAergic and glycinergic neurons, respectively.

**FIGURE 2:**
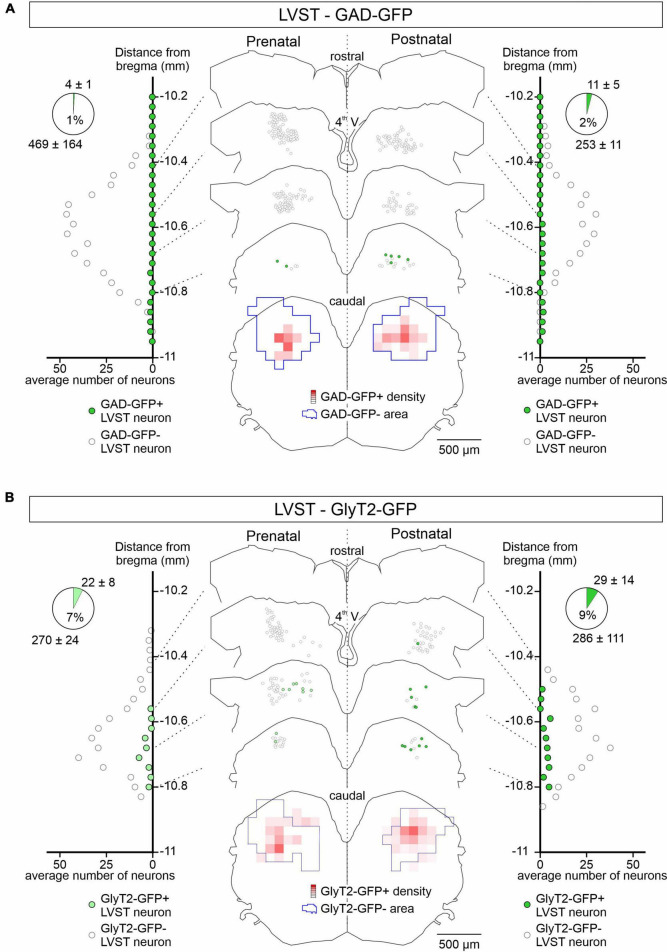
Spatial and temporal organization of GAD-GFP and GlyT2-GFP expression in developing LVST neurons. **(A)** GFP expression in GAD-GFP mice at pre-natal (E15.5 to E18.5; left) and post-natal (P0 to P5; right) stages in LVST neurons identified by retrograde rhodamine dextran amine labeling as in Figure 1. For each side, LVST neurons expressing GFP (green circles) or not (open circles) were localized (representative sections in middle panel) and quantified (lateral diagrams) along the rostrocaudal axis in slices at indicated distances from the bregma (point of junction of the skull’s coronal and sagittal sutures). The section at bottom represents a combined z-projection showing the pre-natal (left) and post-natal (right) distribution densities of GFP-expressing neurons relative to those of non-expressing neurons. Overall cell counts and proportions of the GFP- (upper numbers) and non-GFP labeled neurons (lower numbers) are represented in the pie charts at upper left and right. **(B)** Same layout as in panel **(A)** for GFP expression in LVST neurons from GlyT2-GFP mice. 4th V, fourth ventricle.

GAD67-GFP positive (GABA containing) neurons were almost absent from the LVST neuronal group, representing only between 1% (pre-natal, *n* = 3 preparations) and 2% (post-natal, *n* = 4) of the global LVST population, and when detected, were consistently located medially at the caudal end of the group (Figure 2A). Similarly, GlyT2 GFP-positive (glycine containing) LVST neurons represented only a small fraction (<10%, pre-natal *n* = 4, post-natal *n* = 3 preparations) of the approximately 300 LVST neurons (average from 14 preparations) labeled with retrograde tracer applied at the C1 level (Figure 2B). These neurons were preferentially located from the mid-to-caudal regions of the overall LVST population where they were homogeneously distributed in the dorso-ventral and medio-lateral planes. This expression pattern appeared to be similar at pre- and post-natal stages (compare Figure 2B left, right). These findings thus indicate that the vast majority of LVST neurons are devoid of inhibitory neurotransmitters at early developmental stages, consistent with the situation at late post-natal ages where this group is mainly comprised of excitatory glutamatergic neurons (Du Beau et al., 2012).

**FIGURE 3:**
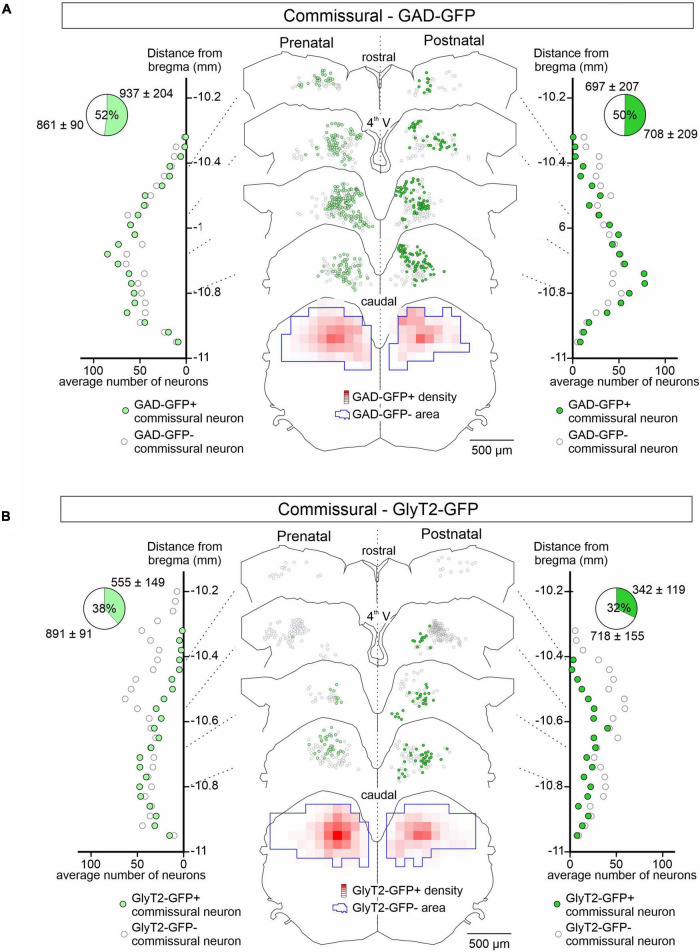
GAD-GFP and GlyT2 expression in developing commissural neurons. Same presentation layout as in Figure 2 for GFP expression (green circles) or not (open circles) in retrogradely dextran-labeled commissural neurons from GAD-GFP **(A)** and GlyT2-GFP mice **(B)** at pre-natal (E15.5 to E18.5; left) and post-natal (P0 to P5; right) stages.

In contrast to the LVST, GAD67-GFP positive neurons represented approximately 50% of the retrograde dextran-labeled vestibular commissural population at both pre-natal (*n* = 4 preparations) and post-natal stages (*n* = 3) along the rostro-caudal axis (Figure 3A). GAD67-GFP neurons were located more medially than non-GFP commissural neurons. Similar to GAD67-GFP neurons, GlyT2-GFP-positive glycinergic neurons also represented a much larger proportion of the commissural population than in the LVST group, being mostly located more medially and caudally in the commissural nucleus and comprising approximately a third (38% pre-natally, *n* = 4 preparations, 32% after birth, *n* = 4) of the commissural population (Figure 3B). This distribution pattern did not appear to change significantly between the pre- and post-natal periods (compare Figure 3B left, right). Finally, it is noteworthy that GAD67-GFP expression in the commissural group tended to be more dorsally located than GlyT2-GFP expression (compare Figures 3A,B), consistent with previous reports suggesting that GABAergic neurons in the MVN are preferentially located in the parvocellular region of the MVN while glycinergic neurons are more often found in the magnocellular region (Tanaka and Ezure, 2004).

**FIGURE 4:**
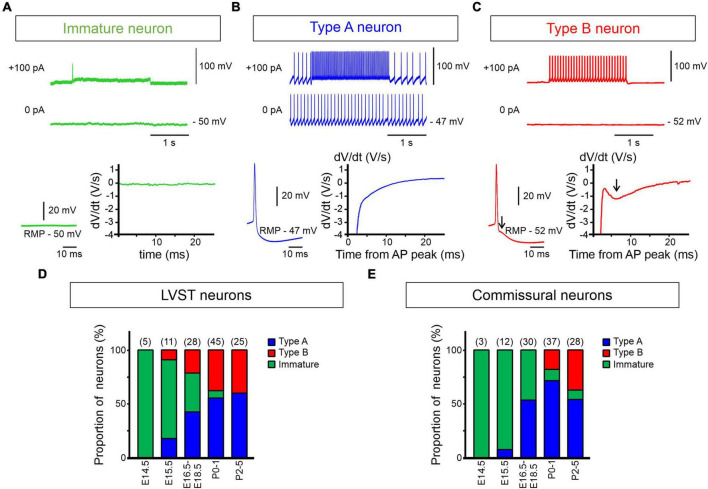
Early emergence of type A and B electrophysiological phenotypes. **(A–C)** Spontaneous and evoked discharge patterns of dextran-labeled perinatal LVST and commissural vestibular neurons. **(A)** A typical immature neuron characterized by the absence of action potentials at resting membrane potential (RMP) and an inability to generate repetitive impulse firing when depolarized (upper traces), found predominantly at pre-natal stages [see panels **(D,E)**]. **(B)** A type A neuron that fired spontaneously at RMP and repetitively at higher frequency in response to a 2 s depolarizing current injection (upper traces). This neuron subtype was characterized by a pronounced monophasic spike after hyperpolarization (bottom left), as shown by the absence of a deflection in the first derivative of the voltage trace (bottom right). **(C)** A type B neuron that was silent at RMP, but produced sustained firing when depolarized. This neuron subtype was characterized by a double spike after hyperpolarization (at arrow in bottom left) that was recognized by a dip in the first derivative of the corresponding voltage trace (at arrow in bottom right). **(D,E)** Proportions of immature (green shading), type A (blue shading) and type B neurons (red shading) from E14.5 to P5 in labeled LVST **(D)** and commissural **(E)** cell populations. In each group, the number of recorded neurons is indicated at the top of each developmental stage.

Together these observations indicate that the vestibular commissural system is already comprised of up to 50% inhibitory cells when the constituent neurons become active at embryonic stages (see below). This percentage is therefore also consistent with reported data on the relative proportions of excitatory and inhibitory commissural neurons at post-natal ages (Straka et al., 2005; Bagnall et al., 2007; Malinvaud et al., 2010).

### Emergence of lateral vestibulospinal tract and commissural neuron subtypes

#### Dual electrophysiological phenotypes

In mice, although vestibular neuron domains are known to be established by E11.5 (Auclair et al., 1999), the electrophysiological properties of vestibular neurons at very early developmental stages have thus far remained unknown. Here, we found that at embryonic age E14.5 all successfully recorded LVST (*n* = 5) and vestibular commissural (*n* = 3) neurons displayed an immature biophysical phenotype (Figures 4A–E), characterized by an inability to produce APs either spontaneously or in response to depolarizing current injection. However, from E15.5 to P5, neurons of both vestibular groups (totaling 114 and 110 recorded LVST and commissural cells, respectively) progressively expressed properties that corresponded to the two general electrophysiological subtypes originally classified by Serafin et al. (1991) for neurons in the adult guinea pig MVN: type A (with a single deep AHP following each AP, Figure 4B) and type B (with a dual-component, early fast and delayed slow AHP, Figure 4C). Correspondingly, the proportion of neurons exhibiting immature properties progressively declined to almost disappear after birth (Figures 4D,E). More precisely, type A and type B LVST neurons were detected from E15.5 onward, with a predominance of type A occurring at subsequent embryonic ages (Figure 4D). From P2 to P5, type A neurons represented 60% of the 25 recorded LVST neurons, which is consistent with previous findings in older mice (Dutia and Johnston, 1998). In contrast, for the commissural group, no type B neurons (out of 45 recorded cells) was detected at pre-natal stages (Figure 4E), while type A neurons consistently represented more than 50% of the recorded neurons from E16.5 onward. It was only after birth that commissural type B neurons appeared, and they then increased to represent 39% of the recorded neurons (*n* = 28) by P2–P5.

#### Morphological correlates

**FIGURE 5:**
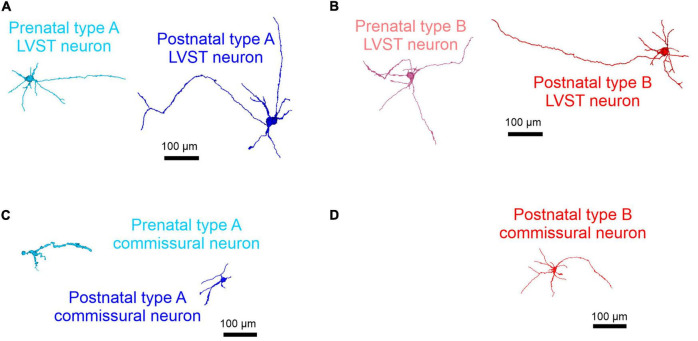
Morphological development of perinatal LVST and commissural vestibular neurons. 3D reconstructions of confocal stacks from representative biocytin-filled neurons that had been previously determined as belonging to the LVST **(A,B)** or commissural populations **(C,D)** by retrograde dextran amine labeling, then identified electrophysiologically as type A (blue shading; **A,C**) or type B (red shading; **B,D**) at pre-natal (E15.5–E18.5, in lighter shading at left of each panel) and post-natal stages (P0–P5, in darker shading at right of each panel). Note that type B commissural vestibular neurons only appeared post-natally.

The morphology of type A and B neurons in the LVST and commissural vestibular subpopulations was visualized by revealing the biocytin present in individual neurons after the tracer’s intracellular diffusion through the patch recording pipette. Confocal 3D reconstructions revealed that both LVST and commissural type A neurons exhibited similar features in terms of cell body size (∼270–300 μm) and number of major neurites (∼4–5) emerging from the soma before and after birth (LVST: Figure 5A and Table 1; pre-natal *n* = 4, post-natal *n* = 8, Mann–Whitney test, *p* > 0.05 for both parameters; Commissural: pre-natal, *n* = 8; post-natal *n* = 10, Figure 5C and Table 2). In contrast, type B LVST neurons consistently exhibited a significantly larger soma after birth (from 127 ± 18 μm^2^, *n* = 3, to 653 ± 132 μm^2^, *n* = 11, Mann–Whitney test, *p* < 0.05). In addition, post-natal type B LVST neurons expressed more soma neurites in general (7 ± 0 neurites, *n* = 11 neurons), which were also significantly more than the neurites of type A neurons after birth (4 ± 0 neurites, *n* = 8 neurons, Mann–Whitney test, *p* < 0.05; Figure 5; Table 1).

**TABLE 1:** Morphological and associated passive membrane properties of types A and B LVST neurons.

	Type A neurons		Type B neurons	
		
	Pre-natal (4)	Post-natal (8)	Pre-natal (3)	Post-natal (11)
Number of soma neurites	5 ± 1	4 ± 0	6 ± 1	7 ± 0 (†)
Soma size (μm^2^)	304 ± 107	276 ± 63	127 ± 18	653 ± 132 (*†)
Input resistance (MΩ)	155 ± 47	190 ± 37	126 ± 28	129 ± 25
Cell capacitance (pF)	53 ± 11	52 ± 8	56 ± 18	64 ± 5

Mann–Whitney tests, (*), *p* < 0.05 for a developmental effect; (†), *p* < 0.05 for a neuronal subtype effect at the equivalent perinatal stage.

Note that the data from these neurons are incorporated in Table 3.

**TABLE 2:** Morphological and associated passive membrane properties of types A and B commissural neurons.

	Type A neurons		Type B neurons	
		
	Pre-natal (8)	Post-natal (10)	Pre-natal (NA)	Post-natal (4)
Number of soma neurites	4 ± 1	4 ± 0		8 ± 1 (‡)
Soma size (μm^2^)	309 ± 67	272 ± 28		429 ± 35 (‡)
Input resistance (MΩ)	326 ± 61	396 ± 90		357 ± 183
Cell capacitance (pF)	31 ± 4 (‡)	30 ± 5 (‡)		40 ± 7 (‡)

Mann–Whitney tests, (‡), *p* < 0.05 for a neuronal subtype effect at the same perinatal stage; (‡), *p* < 0.05 for a vestibular population effect (compare with Table 1); Note that the data from these neurons are incorporated in Table 4.

Similar to the equivalent LVST neurons, post-natal type B commissural neurons (Figure 5D) displayed more soma neurites (8 ± 1, *n* = 4 neurons) than post-natal type A commissural neurons (4 ± 0 neurites, *n* = 10 neurons; Mann–Whitney test, *p* < 0.05; Table 2) and a larger soma (429 ± 35 μm^2^) compared to their type A counterparts (272 ± 28 μm^2^, Mann–Whitney test, *p* < 0.05, Table 2). In accordance with their location in the MVN, where neurons with generally smaller soma size (and therefore less membrane surface area) are typically found (Uno et al., 2003), both types of recorded commissural cells presented a lower membrane capacitance (Mann–Whitney test, *p* < 0.05, Table 2, compare with Table 1) in comparison with their LVST subtype homologs. Overall, type B neurons in the two nuclei exhibited a greater degree of soma neurite complexity compared to type A neurons.

Together, the above data indicate that the two vestibular neuronal populations exhibit an immature electrophysiological phenotype at the earliest embryonic developmental stages examined (E14.5–E15.5). In both functional groups, the type A phenotype, which was found to be consistently larger and structurally more complex in the LVST than the commissural subpopulations, emerges progressively from E15.5 onward, but with little major accompanying change in cell morphology throughout the perinatal period. In contrast, the type B phenotype does not appear at equivalent times in the two vestibular groups. Whereas the first LVST type B neurons, which subsequently increase in soma size and neurite complexity, were found at E15.5, the first commissural type B neurons were detected only at P0, thus indicating a differential ontogenetic time course for this neuronal phenotype according to the functional vestibular population to which it belongs. By the end of the first post-natal week, type A neurons predominate slightly over their type B counterparts in both vestibular nuclei.

### Perinatal development of neuronal membrane properties

**FIGURE 6:**
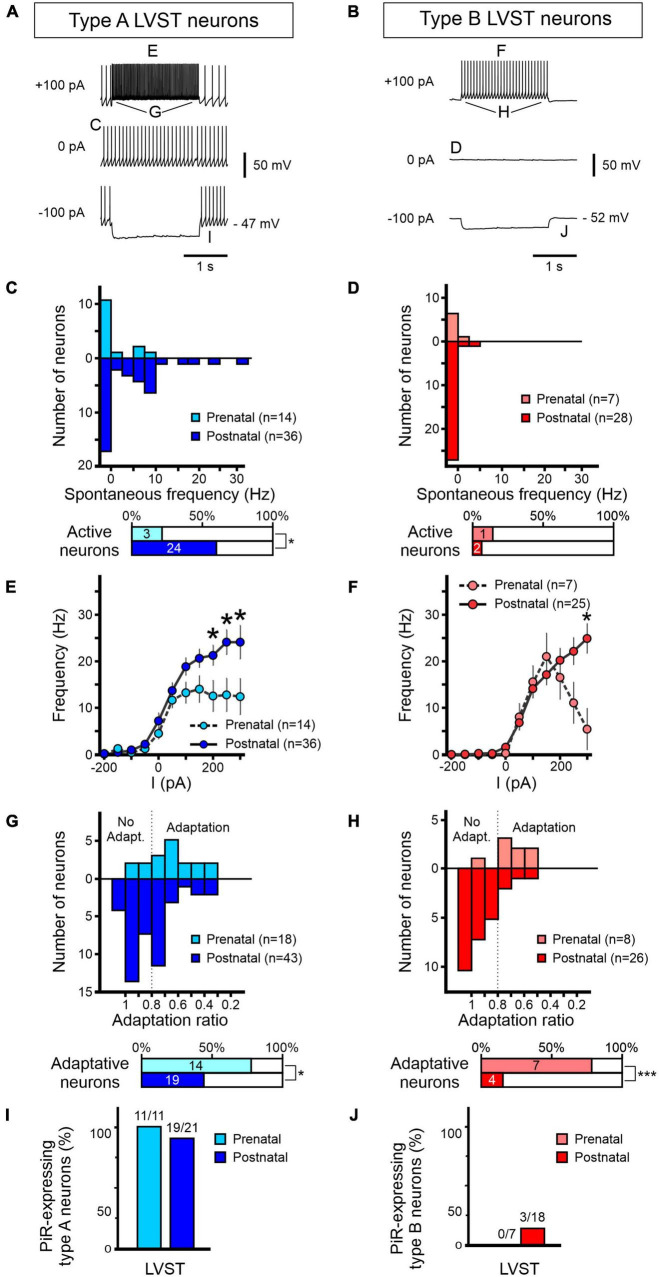
Development of excitability properties of LVST vestibular neurons. Responses to current injections of pre-natal (light blue/red) and post-natal (dark blue/red) retrogradely labeled LVST neurons and subsequently identified as type A **(A,C,E,G,I)** or type B **(B,D,F,H,J)**. **(A,B)** Representative raw traces of LVST neurons at rest (middle) and during 2 s hyperpolarizing (bottom) and depolarizing (top) constant current injections. **(C–J)** Analyses of the pooled responses of the two vestibular neuron subpopulations as indicated by the corresponding letter labeling in panels **(A,B)**. **(C,D)** Distribution of spontaneous firing frequencies classified in bins of 2.5 spikes per sec. Horizontal bar graphs (bottom) represent the proportion of spontaneously active neurons in the corresponding developmental period. **(E,F)** Maximum firing frequencies of the two LVST groups during 2 s constant injected current, increasing in 50 pA steps from –200 to +200 pA. **(G,H)** Adaptation in firing frequency during 2 s, 100 pA depolarizing current injection. The adaptation ratio was calculated from the instantaneous firing frequency at the end of the current pulse over the frequency at the beginning of the pulse. A ratio below 0.8 was considered as adaptive. The percentages of neurons categorized as displaying frequency adaptation are shown in the bottom horizontal bar graphs. **(I,J)** Proportions of neurons expressing a post-inhibitory rebound (PiR) property immediately following release from a 2 s, 200 pA hyperpolarizing current injection [compare the evident PiR in the bottom traces of panel **(B)** with the absence of PiR in the equivalent trace in panel **(A)**]. The number of PiR-expressing neurons *vs* the total number of recorded neurons is indicated above each histogram. ***Mann–Whitney test, *p* < 0.001.

**FIGURE 7:**
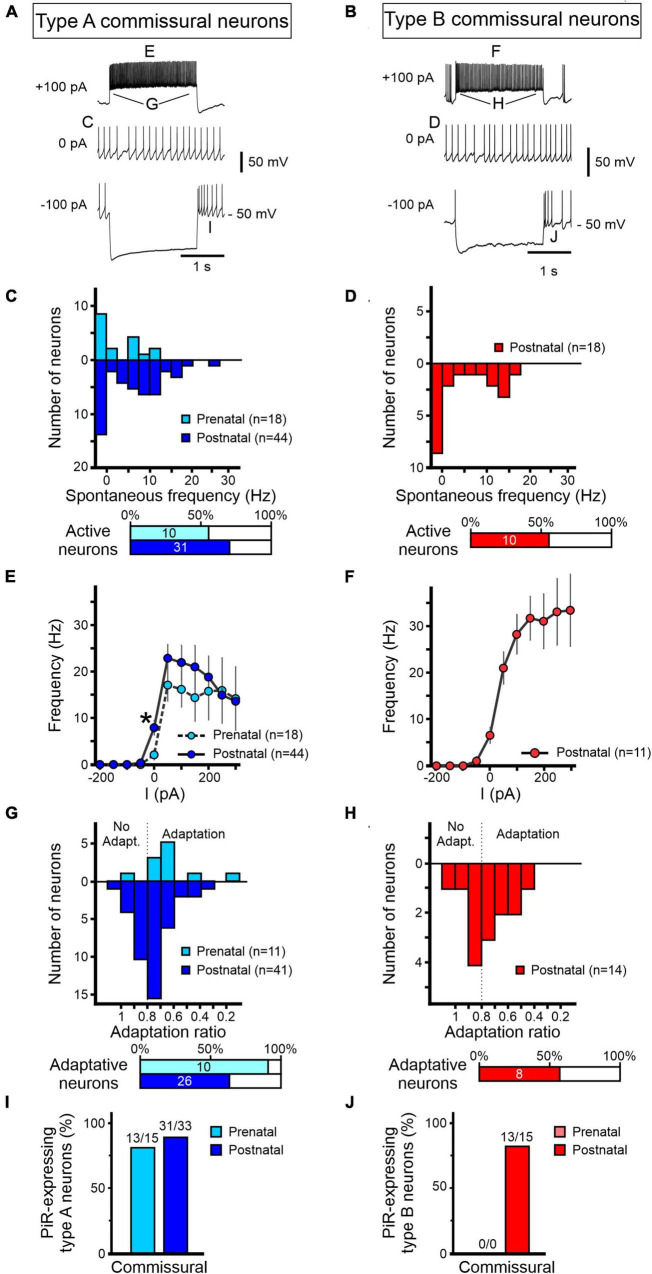
Development of excitability properties of commissural vestibular neurons. Responses to current injections of pre-natal (light blue/red) and post-natal (dark blue/red) identified commissural neurons and characterized as type A **(A,C,E,G,I)** or type B **(B,D,F,H,J)**. **(A,B)** Representative raw traces from commissural vestibular neurons at rest (middle) and during 2 s hyperpolarizing (bottom) and depolarizing (top) constant current injections. **(C–J)** Analysis of the firing properties of type A and B commissural vestibular neurons as described in Figure 6 for LVST neurons. **(C,D)** Distributions of spontaneous firing frequencies at RMP. **(E,F)** Maximum firing frequencies in response to different levels of 2 s current pulse injections. **(G,H)** Firing frequency adaptation during 2 s, 100 pA depolarizing current injection. A ratio of firing rates at pulse onset and offset below 0.8 was considered as adaptive. **(I,J)** Proportions of neurons expressing post-inhibitory rebound (PiR) following release from a 2 s, 200 pA hyperpolarizing current injection. The number of PiR-expressing neurons *vs* the total number of recorded neurons is indicated above each histogram. *Mann–Whitney test, *p* < 0.05.

In a next step, we further explored the perinatal development of the biophysical properties of type A and type B neurons in the LVST and commissural populations. To this end, since no consistent age-related differences were found in the electrophysiological properties of neurons at pre- or post-natal stages in either vestibular population, individual cell data obtained between E15.5 and E18.5 for each A or B subtype were pooled into separate pre-natal groups, as data were obtained from P0 to P5 for two corresponding post-natal groups. The following parameters of neuronal excitability were evaluated by examining the responsiveness of type A (see Figures 6A, 7A for LVST and commissural neurons, respectively) and type B (see Figures 6B, 7B for LVST and commissural neurons, respectively) neurons to step-wise injection of hyperpolarizing and depolarizing currents (from −200 to +200 pA, in 50 pA increments, 2 s duration) under current clamp conditions.

#### Firing properties of lateral vestibulospinal tract neurons

The analyses presented in Figure 6 for the two LVST neuron subtypes during the pre-natal period (from E15.5 to E18.5) were obtained from a total of 18 type A and 8 type B neurons, whereas the two post-natal (P0 to P5) groups comprised recordings from 43 to 28 type A and B neurons, respectively. At resting membrane potential (RMP, i.e., in the absence of injected current), only 21% (3/14; Figure 6C upper panel) of type A LVST neurons were spontaneously active at pre-natal stages with a spontaneous firing frequency ranging from of 0 to 10 Hz (Figure 6C, lower panel). After birth, however, the proportion of spontaneously active type A neurons increased to 60% (24/40) in the post-natal period (Fisher exact test, *p* < 0.05) with a larger frequency range that extended up to 32.5 Hz (Figure 6C).

In contrast, the vast majority of type B LVST neurons remained silent at rest, during both pre- and post-natal stages. Spontaneously active type B neurons represented only 14% of recorded cells before birth and 7% after birth, with a firing frequency that did not exceed 8 Hz (Figure 6D). This difference in intrinsic excitability might be due to a slightly, but significantly, more hyperpolarized RMP measured in type B neurons (pre-natal −52 ± 2 mV, *n* = 7; post-natal −52 ± 1 mV, *n* = 12) compared to type A neurons (pre-natal −46 ± 1 mV, *n* = 11; post-natal −49 ± 2 mV, *n* = 25, Mann–Whitney tests, *p* < 0.05, see Table 3), and is in accordance with RMP differences previously reported in older P5–P30 rat (Dutia and Johnston, 1998). Moreover, consistent with previous studies performed during the first post-natal month (Dutia and Johnston, 1998; Murphy and Du Lac, 2001), AP width in both type A and type B LVST neurons became narrower with age (Mann–Whitney test, *p* < 0.05, Table 3) and type B LVST neurons exhibited an increase in AHP amplitude (Mann–Whitney test *p* < 0.05; Table 3).

**TABLE 3:** Intrinsic membrane properties of LVST neurons.

	Type A		Type B	
		
	Pre-natal (18)	Post-natal (43)	Pre-natal (8)	Post-natal (28)
Input resistance (MΩ)	191 ± 49	202 ± 19	146 ± 15	128 ± 12
Cell capacitance (pF)	69 ± 8	66 ± 12	49 ± 9	60 ± 4
Resting membrane potential (mV)	−46 ± 1	−49 ± 2	−52 ± 2 (‡)	−52 ± 1 (‡)
Firing frequency (Hz), all cells	2 ± 1	5 ± 1	0 ± 0	0 ± 0 (‡)
Firing frequency (Hz), active cells	8 ± 1	9 ± 2	NA	2 ± 1 (*)
AP threshold (mV)	−29 ± 2	−32 ± 2	−32 ± 3	−35 ± 2
AP width (ms)	4.9 ± 0.7	2.7 ± 0.2 (*)	3.5 ± 0.7	2.6 ± 0.5 (*)
AHP amplitude (mV)	16 ± 2	18 ± 1	10 ± 1	18 ± 1 (*)

Mann–Whitney tests, (*), *p* < 0.05 for a developmental effect; (‡), *p* < 0.05 for a neuronal subtype effect at the same developmental stage.

In response to depolarizing current injection, recorded pre-natal type A LVST neurons exhibited a relatively narrow range of AP discharge, increasing proportionally with injected current levels to reach a plateau of 12.5 ± 3.5 Hz with positive currents of +50 pA and above (Figure 6E). After birth, the response curve to current injections up to 300 pA became more linear and reached significantly higher maximal values than before birth for current injections of +200 pA and higher (Mann–Whitney test, *p* < 0.05, Figure 6E). Specifically, the average maximum firing frequency in response to 300 pA current injections increased in type A neurons after birth from 12 ± 4 Hz to 24 ± 4 Hz at pre- and post-natal stages, respectively (Mann–Whitney test *p* < 0.05). Pre-natal type B LVST neurons exhibited a bell-shaped firing response to injected depolarizing current, with the maximum frequency of 21.2 ± 5.1 Hz attained at 150 pA (*n* = 7) before declining progressively with stronger currents (Figure 6F). After birth, the discharge evoked in type B neurons by positive current injection became linear, with the firing frequency in response to the maximum injected current tested (300 pA) reaching a significantly higher level (25 ± 3 Hz compared to 5 ± 4 Hz) in post-natal than pre-natal type B neurons (*n* = 25, Figure 6F, Mann–Whitney test *p* < 0.05).

We next assessed the extent to which LVST neurons display accommodation in their repetitive firing by injecting constant 100 pA depolarizing steps and calculating an adaptation ratio defined as the instantaneous firing frequency at offset over that at the onset of the 2 s injected pulse (Figures 6G,H). Consistent with previous reports at early post-natal stages (Murphy and Du Lac, 2001), in both type A and B LVST neurons, the firing rate adaptation ratio tended to increase with age, i.e., displayed less adaptation, with pre-natal neurons showing a stronger reduction in firing frequency during the test current injection. This developmental effect is evident in Figures 6G,H where the mean adaptation ratios of pre-natal type A and B neurons were 0.65 ± 0.04 and 0.69 ± 0.04, respectively, compared to 0.81 ± 0.03 and 0.95 ± 0.03 for their post-natal equivalents (unpaired *t*-test, *p* < 0.05).

Finally, following hyperpolarizing current injection, vestibular neurons located in the MVN have been previously found to exhibit a transient increase in firing rate, indicative of a PiR property, that is proportional to the level of the prior hyperpolarization (Sekirnjak and du Lac, 2002; Bagnall et al., 2007). In testing for such a property in LVST neurons at perinatal developmental stages, we found that almost all type A neurons expressed PiR in response to a 200 pA hyperpolarizing current injection (Figure 6I), regardless of the pre-natal (11/11) or post-natal (19/21) period. In contrast, no pre-natal (0/7) and a very small proportion (3/18) of post-natal LVST type B neurons expressed a PiR in response to the same injected negative current (Figure 6J).

Together these findings show that type A and type B LVST neurons exhibit different maturational changes in excitability and firing properties over the two perinatal periods studied, presumably commensurate with the respective integrative roles of these two LVST subtypes in the post-natal nervous system (see Section “Discussion”).

#### Firing properties of commissural neurons

Following the same procedures as for LVST neurons, we next examined the development of the firing properties of commissural vestibular neurons, identified by their midline crossing projections toward the contralateral commissural vestibular group (see Figure 1). By performing similar anatomical and functional identification procedures, patch clamp recordings and analysis were made on a total 80 commissural neurons (Figures 7A,B), consisting of 18 pre-natal (E15.5–E18.5) and 44 post-natal (P0–P5) type A commissural neurons, and a solitary group of 18 post-natal type B cells that, as mentioned above (see Figure 4E), did not have detectable pre-natal counterparts.

More than half of the identified commissural neurons were spontaneously active at RMP, regardless of the developmental stage or neuronal subtype (10/18 pre-natal type A neurons; 30/44 post-natal type A neurons, 10/18 post-natal type B neurons; Figures 7C,D). RMP values for both subtypes did not show any significant developmental change (Table 4). Comparable to their LVST equivalents, the range of spontaneous AP frequencies in type A commissural neurons increased from 0 to 12.5 Hz in the pre-natal period to a mean maximum of 27.5 Hz at post-natal stages (Figure 7C). In these neurons, the AP width also became narrower with age (Mann–Whitney tests, *p* < 0.05, Table 4). The range of spontaneous AP frequencies of post-natal type B commissural neurons was narrower (0–17.5 Hz) than commissural type A neurons but noticeably, much larger than their post-natal LVST equivalents (8 Hz; Figure 7D, compare with Figure 6D).

**TABLE 4:** Intrinsic membrane properties of commissural neurons.

	Type A		Type B	
		
	Pre-natal (18)	Post-natal (44)	Pre-natal (NA)	Post-natal (18)
Input resistance (MΩ)	333 ± 47	568 ± 87 (‡)		364 ± 8772 (‡)
Cell capacitance (pF)	25 ± 4 (‡)	30 ± 2 (‡)		37 ± 5 (‡)
Resting membrane potential (mV)	−47 ± 2	−47 ± 1		−49 ± 2
Firing frequency (Hz), all cells	3 ± 1	7 ± 1 (*)		4 ± 1 (‡)
Firing frequency (Hz), active cells	6 ± 1	9 ± 1		8 ± 2
AP threshold (mV)	−30 ± 2	−32 ± 2		−35 ± 2
AP width (ms)	5.1 ± 0.5	3.5 ± 0.2 (*)		2.7 ± 0.3 (‡)
AHP amplitude (mV)	16 ± 2	18 ± 1		15 ± 2

Mann–Whitney tests, (*), *p* < 0.05 for a developmental effect; (‡), *p* < 0.05 for a neuronal subtype effect at the same developmental stage; (‡), *p* < 0.05 for a vestibular population effect (compare with Table 3).

Type A commissural neurons exhibited a narrow dynamic firing range in response to current injections regardless of the developmental stage, plateauing at a mean of 15.6 ± 0.8 Hz for depolarizing currents above 50 pA (pre-natal *n* = 18, post-natal *n* = 44, Figure 7E). Their maximum frequency in response to current injections (at ∼50 pA) was 30 ± 6 Hz and 36 ± 4 Hz for pre- and post-natal neurons, respectively. In contrast, the response to current injections in post-natal type B commissural neurons was more linear, although plateauing somewhat at the highest values of injected current (*n* = 11, Figure 7F).

During depolarizing current injections, commissural type A neurons displayed a firing adaptation ratio that increased through perinatal development, with pre-natal neurons showing a stronger reduction in firing rate during current injection (0.64 ± 0.06) compared to their post-natal equivalents (0.75 ± 0.02, unpaired *t*-test, *p* < 0.05, Figures 7G,H). It is noteworthy that whereas a reported feature of type B neurons located in the MVN of the adult CNS is their marked decrease in firing frequency during depolarizing current injections (Straka et al., 2005), we found that only about half (8/14) of post-natal commissural type B neurons expressed such an adaptation. In response to hyperpolarizing current injections, almost all type A commissural neurons expressed a PiR after a −200 pA current step, regardless of their development stage (Figure 7I), indicating the PiR property is present in type A neurons from embryonic ages onward. Similarly, most post-natal type B commissural neurons (13/15) exhibited a PiR property (Figure 7J).

Overall, in contrast with LVST neurons, our findings indicate that no major perinatal developmental changes occur in the basic excitability properties of the two types of commissural neurons, except a slightly increased accommodation in firing responsiveness to membrane depolarization and other than the fact that type B neurons only emerge at post-natal stages.

#### Current-voltage relationships of lateral vestibulospinal tract and commissural neurons

In a further step, we asked whether the excitability properties of the two types of LVST and commissural vestibular neurons at perinatal stages could be attributed to the expression patterns of specific sets of voltage-gated conductances. To this end, under voltage clamp conditions, we first determined steady levels of membrane current in LVST and commissural neurons measured at the end of responses to 400 ms command voltage steps from a holding potential of −40 mV to between −100 and +30 mV.

**FIGURE 8:**
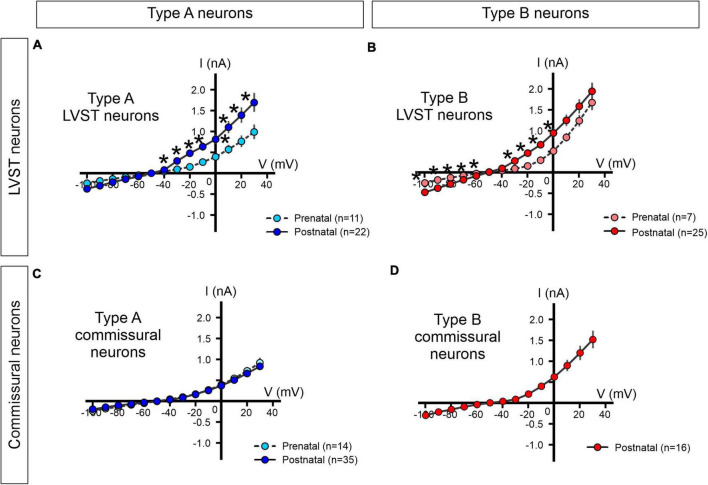
Steady-state current-voltage (I/V) relationships of perinatal LVST and commissural vestibular neurons. The voltage-dependence of steady-state membrane currents was assessed under voltage-clamp where pre-natal (light blue/red) and post-natal (dark blue/red) neurons were clamped at increasing voltages from –100 to +30 mV from a –40 mV holding potential. Measurements were made at the end of each 400 ms command voltage step. **(A,B)** Average I/V plots for pre- and post-natal type A and type B LVST neurons, respectively. **(C)** I/V curves for pre- and post-natal type A commissural neurons. **(D)** I/V curve for post-natal type B commissural neurons. *Mann–Whitney test *p* < 0.05.

The steady-state current–voltage relationships (I/V curves) obtained for LVST type A neurons at both pre-natal (*n* = 11) and post-natal (*n* = 22) stages expressed a non-linear deviation, especially at depolarized membrane potentials, indicative of a net dominance in the activation of voltage-dependent outward currents (Figure 8A). At post-natal stages, this outward rectification was more pronounced such that at all depolarized potentials currents, the mean slope of the I/V curve was significantly greater than at pre-natal stages (Mann–Whitney tests, *p* < 0.05), therefore indicating a net increase in these steady-state outward membrane conductances after birth. The I/V curves constructed for type B LVST neurons also expressed a non-linear slope at imposed depolarized membrane potentials (Figure 8B). However, in the post-natal period, type B LVST neurons (*n* = 25) expressed significantly larger currents at all negative potentials, although not at the most positive potentials when compared to pre-natal equivalents (*n* = 7, Mann–Whitney tests).

Although commissural neurons also exhibited outward rectification in steady-state levels of membrane current at depolarized membrane potentials (pre-natal *n* = 14, post-natal *n* = 51; Figures 8C,D), the I/V curves of type A commissural neurons remained virtually identical at pre- and post-natal stages (Figure 8C), thereby suggesting an absence of developmental change in the underlying voltage-gated currents. It is noteworthy, finally, that type B commissural neurons, which only appear after birth, exhibited a significantly stronger rectification than post-natal type A commissural neurons (*n* = 16, Mann–Whitney test, *p* < 0.05, Figure 8D, compare with Figure 8C).

#### Other voltage-gated conductances

Amongst the best characterized individual conductances contributing to vestibular neuron function, both the persistent sodium current (INaP) and the hyperpolarization-activated current (Ih) are known to underlie AP genesis and PiR firing (Sekirnjak and du Lac, 2002; Bagnall et al., 2007). Since these two conductances are not activated by the constant step-command protocol used for constructing the steady-state I/V relationships described above, we investigated the developmental expression of these currents in LVST neurons using specific command protocols that lead to their activation.

**FIGURE 9:**
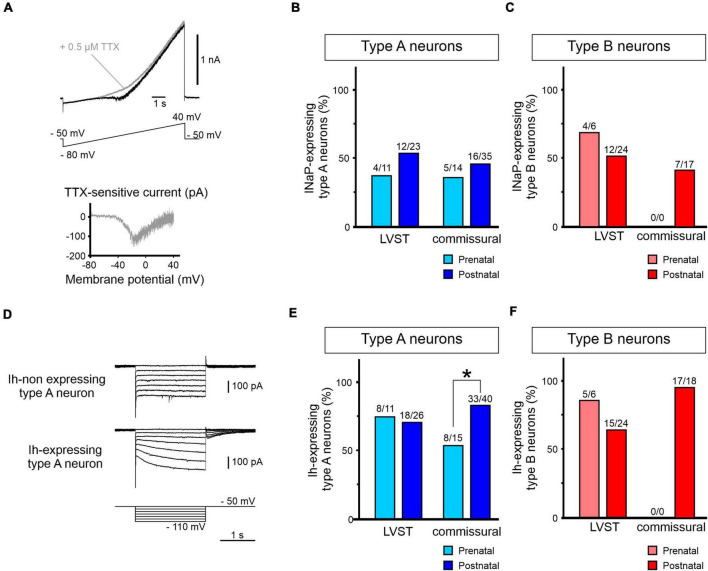
Perinatal expression of INaP and Ih in perinatal LVST and commissural vestibular neurons. **(A)** Activation of the persistent sodium current (INaP) by an increasing voltage ramp depolarization from –80 to +40 mV over 9 s (lower trace in upper panel). INaP typically appears as an inward current inflection developing from approximately –40 mV (black trace in upper panel). INaP is sensitive to the sodium channel blocker TTX (0.5 μM, gray trace), allowing the current to be isolated by subtracting steady current levels before and after TTX exposure (lower panel). **(B)** Group data showing proportions of type A LVST and commissural neurons expressing INaP before (light blue) and after birth (dark blue). **(C)** Equivalent group data (in light and dark red) for the presence of INaP in type B LVST and commissural neurons. **(D)** Voltage clamp recordings from different vestibular neurons without hyperpolarization-activated (h) currents (upper traces) or with Ih (middle traces), as revealed by the characteristic current sag during 2 s constant voltage steps imposed in increments of 10 mV from –110 to –50 mV (bottom traces). **(E)** Group data showing proportions of type A LVST and commissural neurons expressing Ih before (light blue) and after birth (dark blue). **(F)** Equivalent group data (in light and dark red) for the presence of Ih in type B LVST and commissural neurons. *Fisher exact test *p* < 0.05.

First, the NaP current can be activated with a slowly depolarizing 9 s ramp from −80 to +40 mV (Magistretti and Alonso, 1999; Figure 9A), thus appearing as an inward current inflection that develops at ramp increases above −40 mV. In a subset of recorded neurons (*n* = 4), the identity of the current was further confirmed by its sensitivity to 0.5 μM TTX (Figure 9A, upper panel), with the current’s I/V relation after isolation being plotted by digitally subtracting the traces recorded before and after exposure to TTX (Figure 9A, lower panel).

The proportion of tested LVST neurons expressing INaP was not statistically different before and after birth in the two A and B subtypes [36% (4/11) and 52% (12/23) of pre- and post-natal type A, respectively; Figure 9B, left; 66% (4/6) and 50% (12/24) of pre- and post-natal type B, respectively; Fisher exact test, *p* > 0.05; Figure 9C, left]. Moreover, less than 50% of commissural neurons was found to express INaP [36% (5/14) and 46% (16/35) of pre- and post-natal type A, respectively; Figure 9B, right; 49% (23/47) post-natal type B; Figure 9C, right], and here again, there was no evidence for significant cell-wide changes in the current’s perinatal expression (Fisher exact test, *p* > 0.05).

Second, the presence of the Ih current was assessed by the development of an instantaneous and slowly activating inward current during hyperpolarizing voltage steps (Figure 9D). In type A LVST neurons, around 70% of pre-natal (8/11) and post-natal (18/26) neurons expressed detectable Ih (Fisher exact test, *p* > 0.05; Figure 9E, left). Similarly, recorded type B LVST neurons expressed Ih in similar cell proportions and in a development-independent manner [pre-natal 83% (5/6), post-natal 62.5% (15/24), Figure 9F, left; Fisher exact test, *p* > 0.05]. However, whereas 53% of pre-natal commissural type A neurons (8/15) expressed an Ih conductance, this proportion increased significantly to 83% (33/40) of post-natal type A neurons (Fisher exact test, *p* < 0.05, Figure 9E, right). This relatively high expression of the Ih conductance was also observed in post-natal type B commissural neurons [94% (17/18); Figure 9F, right].

Taken together, these data show that although the firing behavior and, as yet unidentified, voltage-dependent properties of LVST neurons undergo changes during the perinatal period, the unaltered expression of the NaP and h currents in both type A and B phenotypes between E15.5 and P5 indicate that these two inward conductances do not contribute to this plasticity. In direct contrast, despite the absence of major maturational alterations in the basic membrane properties of commissural neurons, our findings point nonetheless to a developmental increase in the numbers of neurons with Ih, with the vast majority of cells expressing this conductance by post-natal stages.

## Discussion

In this study, we provide data on the early developmental processes related to the neurotransmitter, morphological and electrophysiological properties of central vestibular nuclei neurons constituting two functionally distinct neuronal groups, the LVST and vestibular commissural subpopulations. Our investigation, which addressed a developmental period that *hitherto* has remained largely undescribed, examined neuronal populations that were identified unambiguously on the basis of their axonal projections and somata labeling, rather than solely according to the brainstem vestibular nuclei in which they are located, as in most other previously reported studies.

Our major findings are that the A and B neuronal phenotypes are already distinguishable during early vestibular ontogeny according to AP and AHP profiles as described in the literature for sub-adult and mature rodents (Serafin et al., 1991; Johnston et al., 1994; Beraneck et al., 2003; Straka et al., 2005; Eugène et al., 2011). However, the two functional subgroups do not follow the same maturational time course. Type A neurons already begin to emerge from electrophysiologically immature precursors in both the LVST and the commissural group at E15.5. In contrast, functional type B neurons appear differentially between LVST and commissural groups. Whereas LVST type B neurons are also present at E15.5, like type A neurons, commissural type B neurons do not appear until post-natal (P0–P1) stages. This suggests that the type B phenotype could be differentially involved in the early maturation of distinct functional vestibular subgroups as shown here for LVST and commissural neurons. Subsequently, the respective proportions of each cell type change throughout the perinatal period, with the appearance of both A and B subtypes increasing as the immature phenotype diminishes. In the two vestibular populations, moreover, the distribution of inhibitory neurotransmitter phenotypes appears to be fully established as soon as neurons of the two vestibular populations become active (and presumably on reaching their projection sites), with GABA/glycinergic cells displaying within-group proportions similar to those found in older post-natal stages. In contrast, neuron morphologies, including soma size, axonal projections and dendritic trees, especially of LVST neurons, generally continue to develop throughout the perinatal period. While the electrophysiological properties of type A commissural neurons appear to remain stable during early development, type A LVST neurons exhibit significant developmental maturation in several of their discharge properties. The same maturation time course was observed for type B LVST neurons, suggesting a common developmental feature to all neuronal phenotypes in the LVST group. These include an increase in the dynamic range of firing responsiveness to membrane depolarization and a general decrease in spike frequency adaptation. Thus, overall our results show that the early functional maturation of vestibular neuron properties differ depending upon the cell type and the specific vestibular circuitry to which the constituent elements belong. Interestingly, a previous study reported that a majority of neurons from the LVN in adult guinea pig (Uno et al., 2003), containing mainly spinal projecting neurons, were type B neurons, in disagreement with our present findings (∼50% of type A). Although this difference could be explained by species variability, the study of Uno et al. (2003) focused solely on Deiter neurons (with large somata) without including other LVN spinal projecting neurons. On the other hand, the present study was made on LVST neurons identified by spinal retrograde labeling, and therefore presumably included both Deiter and non-Deiter neurons, and regardless of size. An additional explanation for the different findings of the two studies is that type A neurons are transiently expressed in the LVN population during early development (before P5), but then transform to an exclusive type B phenotype during later maturation. This latter possibility cannot be excluded when neuron classification is based on spike shape alone, and especially during early development.

### Perinatal development of neurotransmitter phenotypes

Our data from GFP-expressing transgenic mice lines showed that both GABAergic and glycinergic neurons are present from embryonic stages in the LVST and vestibular commissural populations. Moreover, their respective proportions in the two populations, as well as their patterns of rostro-caudal and dorso-ventral distribution, remain stable over the first 5 days after birth. More specifically, in the commissural group, inhibitory neurons represent up to 52% of the total population, corresponding to previous observations at sub-adult stages and in different species that this group is composed of approximately equally represented subpopulations of excitatory and inhibitory neurons (Bagnall et al., 2007; Malinvaud et al., 2010). In addition, we found that commissural GAD67-GFP positive neurons were located in the MVN more dorsally than commissural GlyT2-GFP positive neurons, which is also consistent with previous reports in rat indicating that GABAergic neurons predominate in the parvocellular region of the MVN, whereas glycinergic neurons are more often located in the magnocellular region (Tanaka and Ezure, 2004).

In contrast, we found that only a very limited proportion (<10%) of LVST neurons contain an inhibitory transmitter, being mostly glycine. This in turn implies that at very early stages of development, the vast majority of LVST neurons are excitatory in function, which is in accordance with the literature obtained at later stages of maturation where the LVST group is predominantly glutamatergic (Du Beau et al., 2012). Interestingly, in the rat LVN where the majority of LVST somata are found, most GABAergic neurons also contain GLYT2 mRNA (Tanaka and Ezure, 2004), which raises the possibility, at least for the LVST population, that GABA and glycine are co-localized within a subgroup of the same neurons. On the other hand, a developmental switch from GABAergic to glycinergic neurotransmission has also been reported in other regions of the central nervous system (Kotak et al., 1998; Gao et al., 2001; Nabekura et al., 2004). Further work is therefore needed to determine the extent to which GABA and glycine are contained in the same vestibular neurons, and whether such a spatial overlap reflects a developmental transition from one neurotransmitter system to the other or a capability for actual co-transmission as a property of synaptic function.

Such an examination of the neurotransmitter phenotype of vestibular neurons is of particular relevance since a rebalancing of the synaptic actions of vestibular pathways contributes to so-called vestibular compensation, a form of neuronal plasticity that emerges in response to a vestibular dysfunction [(Precht et al., 1966; Smith and Curthoys, 1989; Ris et al., 1995; Ris and Godaux, 1998; Bergquist et al., 2008) and for reviews see (Lambert and Straka, 2012; Le Ray and Guayasamin, 2022)]. From a functional perspective also, it is noteworthy that at early developmental stages similar to those examined here, chloride-mediated synaptic signaling can exert excitatory rather than inhibitory influences due to an immature chloride gradient in brainstem neurons (Ehrlich et al., 1999; Kakazu et al., 1999). Consequently, caution must be taken in assigning an inhibitory function to GABA/glycine containing vestibular neurons at perinatal ages. Nevertheless, although further experiments are required to establish when chloride-mediated neurotransmission becomes inhibitory in the vestibular nuclei, our data indicate that the neurotransmitter phenotype(s) required for synaptic inhibition in the post-natal CNS are already established at the earliest stages when vestibular neurons become bio-electrically functional.

### Perinatal development of electrophysiological properties

We also found that neurons belonging to the two vestibular populations exhibit maturational changes in their electrophysiological properties from E14.5 onward. Most notably, type B commissural neurons displayed a belated acquisition of their particular electrophysiological phenotype, which in contrast to type A commissural neurons and both type A and B LVST neurons, did not emerge pre-natally but were detected exclusively after birth. While more neurons of the two vestibular groups exhibited spontaneous impulse discharge after birth–with the exception of type B LVST neurons that were almost always silent at rest throughout the perinatal period (see below)–the majority of recorded vestibular neurons remained inactive in the absence of depolarizing current injection. This contrasts with mature vestibular neurons that for the most part fire spontaneously (Murphy and Du Lac, 2001; Uno et al., 2003; Bagnall et al., 2007).

To further assess the emergence of vestibular neuron discharge properties, we investigated the expression of two specific conductances that are typically involved in setting steady-state membrane potential and the intrinsic pacemaker ability of neurons to fire spontaneously: the hyperpolarization-activated cation current Ih and the slowly inactivating persistent sodium current INaP (Yamada-Hanff and Bean, 2013, 2015). In the present study, however, a developmental dynamic involving either conductance, at least in the two studied vestibular neuron populations studied, does not appear to underlie the emergence of a spontaneous pacemaker-like capability. Therefore, notwithstanding the possibility that developmental changes in the actual densities of Ih and/or INaP lead to alterations in the firing behavior of individual neurons (Burton et al., 2008), our present data suggest that early in development, the induction of pacemaker-like activity in vestibular neurons relies on other ionic conductances that remain to be identified. Moreover, it has been reported that the tonic firing capability of vestibular neurons undergoes post-natal development that is both temporally and spatially variable, with the rostral MVN region exhibiting earlier maturation than its more caudal counterpart (Dutia et al., 1995). Since most of our recordings were made in the rostral part of the vestibular nuclei, it is unlikely that the apparent progressive acquisition of the spontaneous pacemaker-like activity we describe here is in fact due to spatial variations in our sites of recording.

One of the most marked electrophysiological features we found to evolve after birth was the firing rate sensitivity of vestibular neurons to depolarizing current injection. All neurons recorded at pre-natal stages exhibited rapidly plateauing response to constant injected positive currents which was associated with a strong adaptation in firing rate during the course of the current injection. Such a property suggests that embryonic vestibular neurons are yet unable to integrate and process a large range of afferent synaptic information. After birth, however, the responsiveness, notably of LVST neurons to imposed depolarization, became more proportional to current level, exhibiting a near linear initial discharge response and a reduced firing rate adaptation to maintain stimulation. It is likely that the increased capacity to fire in a sustained, non-adapting manner is related to the increase in outward current that leads to a more rapid repolarization after a spike, minimizing the degree of Na channel inactivation, thereby allowing continued spiking, similar to what was found in more mature MVN neurons (Gittis and du Lac, 2008). Interestingly, these maturational processes immediately after birth occur concomitantly with a progressive acquisition of postural control and locomotion (Clarac et al., 1998; Jamon and Clarac, 1998; Lelard et al., 2006), that in turn require the effective integration of a large range of synaptic inputs. In type A commissural neurons, however, the predominant cell type we found in the MVN, the discharge response to current injection saturated and plateaued even at low current amplitudes after birth, consistent with earlier evidence that the maturation of spike generation in the mouse MVN occurs during the second week of life, just before eye-opening (Murphy and Du Lac, 2001). This finding is also commensurate with the idea that the maturation of vestibular afferent pathways themselves is likely to participate in establishing the adult encoding properties of their vestibular neuron targets (Beraneck et al., 2004; Straka et al., 2005; Beraneck and Lambert, 2020), and even potentially in specifying the latter’s A or B phenotype earlier in development.

A further striking observation in the present study was the unique bioelectrical behavior displayed by type B LVST neurons compared to the other recorded vestibular neurons. Almost all neurons of this subtype remained silent at RMP, even after birth, and did not show firing rate adaptation with sustained depolarizing current injections. In addition, these neurons usually did not display any rebound potentiation following hyperpolarizing current injections, despite their widespread expression of INaP and Ih. This suggests that early in development, type B LVST neurons are still very limited in their ability to process synaptic inputs, but would be more adapted for the integration and transmission of transient or high-frequency afferent information (Murphy and Du Lac, 2001). Such a maturational delay in processing capability by LVST neurons compared to commissural neurons is consistent with the initial emergence of a head stabilizing ability (via an earlier maturation of the commissural pathways), followed by the control of the trunk and limbs [via the maturation of the vestibulo-spinal tracts (Beraneck et al., 2014; Lambert et al., 2016)]. An alternative explanation is that LVST neurons in slice preparation are devoid of neuromodulatory or other inputs that might be active in the intact animal. Evidence in support of this idea comes from an efference-copy type activity found in LVN neurons of cat (Orlovsky, 1972; McCall et al., 2016), pigeon (McArthur and Dickman, 2011), and zebrafish (Barrios et al., 2020), as well as the observation that LVN neurons are strongly responsive to orexin (Zhang et al., 2011). Thus, the low spontaneous firing rates such as we observed might not necessarily be indicative of a low responsiveness overall.

In conclusion, while our present work remains mostly descriptive, it does extend knowledge on the early maturation of vestibular neurons during late embryonic and early post-natal development, by unraveling major changes associated with the progressive acquisition of their adult electrophysiological phenotype. Significantly, the changes observed here parallel the maturation of postural reflexes and likely also the development of vestibular afferent activity and/or synaptic efficacy. Indeed, consistent with this latter possibility, we have recently found that *in vitro* stimulation of the vestibulocochlear nerve elicits AP firing in only approximately 30% of pre-natal LVST and commissural neurons, whereas the same afferent nerve stimulation is almost 100% effective after birth (Unpublished observations). On the other hand, an alteration of vestibular inputs by deafferentation has been shown to alter the intrinsic electrophysiological properties of vestibular neurons in the sub-adult and mature rodent nervous system (Beraneck et al., 2003, 2004; Camp et al., 2006; Eugène et al., 2007). These observations thus further indicate that the maturation of vestibular afferents and their functional efficacy closely matches the developmental processes underlying the acquisition and maintenance of the electrophysiological properties of central vestibular neurons (for a review, see Beraneck and Lambert, 2020). Further work is now needed to determine precisely the role played by the connectivity, synaptic strength, and neurotransmitter characteristics of these afferents in governing this post-synaptic plasticity.

## Data availability statement

The raw data supporting the conclusions of this article will be made available by the authors, without undue reservation.

## Ethics statement

The animal study was reviewed and approved by University of Bordeaux Ethics Committee.

## Author contributions

CD and LC performed the experiments. CD performed the analysis. CD, FL, and MT-B designed the study. CD, JS, FL, and MT-B wrote the manuscript. All authors contributed to the article and approved the submitted version.
